# Stable Near-Infrared Photoluminescence of Hexagonal-Shaped PbS Nanoparticles with 1-Dodecanethiol Ligands

**DOI:** 10.3390/ma17102380

**Published:** 2024-05-16

**Authors:** Tsair-Chun Liang, Hsin-Yu Su, Kasimayan Uma, Sih-An Chen, Zhi-Chi Deng, Tzung-Ta Kao, Chun-Cheng Lin, Lung-Chien Chen

**Affiliations:** 1 Institute of Photonics Engineering, National Kaohsiung University of Science and Technology, Kaohsiung 824, Taiwan; 2 Organic Electronics Research Center, Ming Chi University of Technology, New Taipei City 243, Taiwan; 3 Department of Electro-Optical Engineering, National Taipei University of Technology, Taipei 106, Taiwan; 4 Department of Mathematic and Physical Sciences, General Education Center, R.O.C. Air Force Academy, Kaohsiung 820, Taiwan

**Keywords:** PbS nanoparticles, thio-ligand, NIR region, hydrophilic

## Abstract

In this study, lead(II) sulphide (PbS) nanoparticles of varying particle sizes were synthesized using the hot injection method, employing 1-octadecene (ODE) as a coordinating ligand in conjunction with oleylamine (OAm). This synthesis approach was compared with the preparation of hexagonal-shaped nanoparticles through the ligand of 1-Dodecanethiol (DT), resulting in DT-capped PbS nanoparticles. The prepared nanoparticles were characterized using multiple techniques including photoluminescence (PL), transmission electron microscopy (TEM), X-ray diffraction (XRD), and Fourier-transform infrared spectroscopy (FT-IR). The condensation reaction of DT ligands led to various nanoparticles within the range of 34.87 nm to 35.87 nm across different synthesis temperatures (120 °C, 150 °C, 180 °C, 210 °C, and 240 °C). The PbS with DT ligands exhibited a highly crystalline and superhydrophilic structure. Interestingly, near-infrared (NIR)-PL analysis revealed peaks at 1100 nm, representing the lowest-energy excitonic absorption peak of PbS nanoparticles for both ligands. This suggests their potential utility in various applications, including IR photoreactors, as well as in the development of non-toxic nanoparticles for potential applications in in vivo bioimaging.

## 1. Introduction

Recently, there has been a notable surge in the study of semiconductor nanoparticles, particularly those of small sizes ranging from 20 nm to 50 nm. They have garnered significant attention due to their distinct physicochemical properties compared to bulk materials or larger particles [1,2]. The ability to manipulate morphology and size enables the tuning of the broad near-infrared band edge into the ultraviolet range. These distinctive properties have ignited interest in a multitude of applications, spanning from solar cells to near-infrared (NIR) region communication, imaging sensors, nonlinear optics, and light-emitting diodes [3,4,5,6]. Recently, there has been interest in synthesizing lead(II) sulfide (PbS) nanoparticles, primarily owing to their classification within the IV-VI semiconductor. This heightened interest may be attributed to several factors, including their notably large exciton Bohr radius, high dielectric constant, and relatively small band gap of approximately 0.41 eV. Moreover, a particularly appealing characteristic of PbS nanoparticles is their band gap tunability, spanning from as low as 0.3 eV to 5.2 eV, rendering them versatile for a myriad of applications [7,8,9,10].

In today’s landscape, there is a demand for NIR detection across various sectors including automation, the military, and healthcare. However, a persistent challenge persists: NIR detection remains expensive, requiring multifaceted production equipment and encountering difficulties in integration with traditional III–V semiconductors. Likewise, the toxicity of NIR nanoparticles is a significant concern, as they may contain metals such as mercury, lead, tellurium, and selenium, which are known for their chronic toxicities when introduced into living organisms. Therefore, there is a critical need for the development of new NIR quantum dots or nanoparticles that exhibit a unique optical window for tissue penetration while maintaining low toxicity levels. In this context, PbS nanoparticles have emerged as promising candidates for commercial NIR detection due to their exceptional light absorption capabilities, cost-effectiveness, and tunable band gap, while having relatively low toxicity due to their low water solubility. The bulk material form of PbS was initially developed several decades ago, around 1940, and since then, PbS nanoparticles have seen growing utilization in the realm of NIR detectors [11]. In 2005, the fabrication of PbS photoconductors marked a significant milestone, allowing for comparison with InGaAs materials [12].

While a wide array of nanoparticles has been synthesized using various chemical methods aimed at modifying their optoelectronic properties by controlling their morphology, limited attention has been given to achieving facile control over their particle size and shape. Consequently, solvent-based techniques such as sol–gel and solvothermal methods, which prioritize low-temperature processes, stable nanomaterials, and extended lifetimes, have become prevalent among researchers [13,14]. Many studies have explored methods involving coordinated solvents to produce non-toxic materials, notably employing cetyltrimethylammonium bromide (CTAB) and long-chain alkylamines such as hexadecylamine and oleylamine [15,16]. These solvents are favored for their ability to yield materials of superior quality and high efficiency with prolonged lifetimes. Further, these synthetic techniques can be used to achieve high-quality PbS nanoparticles, enabling the fabrication of materials with diverse sizes and shapes using metal sulfide.

This study delved into variations induced by reaction time and temperature, while employing robust capping agents to stabilize the materials. Conversely, another study has highlighted employing small organic molecules as capping agents for PbS nanoparticles, leading to agglomeration and oxidation processes. Various capping agents, such as glucose, oleylamine, thiocyanate, halide, and mercaptopropionic acid, have been explored for nanoparticle preparation, serving to passivate the surface [17,18,19,20,21,22,23]. For a given ligand, the order–disorder phase transition in PbS, triggered by interactions with capping ligands, enhances the conductivity of PbS nanoparticles, paving the way for advancements in optoelectronic devices [24]. However, long-chain ligands have been found to restrict charge transport, while shorter ligands undergo condensation reactions and have structures which exhibit higher conductivity, facilitating electron transfer and improving electron coupling between nanoparticles within the optimal reaction temperature range of 200–300 °C. Thioacetate is specifically employed in the synthesis of CdS quantum dots, resulting in a thin-film transistor (TFT) structure exhibiting a remarkably high field-effect mobility of 8 cm^2^ V^−1^ S^−1^ [25]. In this research, thiol groups participated in condensation reactions forming -S- linkages on the quantum dot surfaces, yielding a densely packed solid. Notably, this arrangement led to a short interatomic distance of approximately 4.15 Å, enhancing electronic coupling and dipole–dipole interactions among the quantum dots, thus significantly improving device performance.

In this study, the PbS nanoparticles were capped with ODE-OAm, which resulted in cubic-shaped structures across temperatures ranging from 120 °C to 240 °C. Further, we demonstrate that PbS nanoparticles synthesized using DT ligands lead to the formation of hexagonal shapes with similar particle sizes. The distinctive hexagonal shape of the PbS particles derived from DT molecules is attributed to the strong bonding occurring predominantly on the (111) plane of the PbS. Additionally, the alkyl chain of the DT ligand effectively passivated the sulfur atoms, thereby contributing to the peak intensity observed in the near-infrared region of 1100 nm. Our results suggest that PbS nanoparticles capped with DT exhibit better structural stability under ambient conditions compared to those capped with ODE-OAm.

## 2. Materials and Method

Oleic acid (OA) and 1-octadecene (ODE) were obtained from TCL Technology (Huizhou, China), while oleylamine (OAm) was sourced from Acros Organics (Taipei City, Taiwan). Sodium diethyldithiocarbamate trihydrate (Na(DDTC)), lead(II) nitrate (Pb(NO_3_)_2_), n-Octane, dimethylformamide (DMF) ethanol, and 1-dodecanethiol (DT) were acquired from Sigma Aldrich (St. Louis, MO, USA). All materials were used as received without further purification.

**Figure 1:**
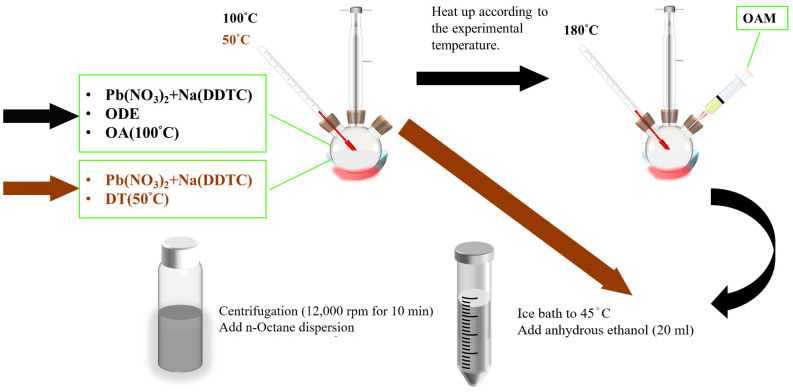
A schematic diagram detailing the synthesis process of PbS nanoparticles, highlighting the addition of ODE-OAm and DT.

The synthesis of PbS nanoparticles utilizing ODE-OAm and DT ligand structures involved the initial combination of 2.2531 g of Pb(NO_3_)_2_ with 1.6560 g of Na(DDTC) in 6 mL of ODE. This mixture was then supplemented with 3 mL of OA and subjected to degassing at 100 °C for one hour before reaching experimental temperatures (120 °C, 150 °C, 180 °C, 210 °C, and 240 °C). After temperature stabilization, 3 mL of OAm was introduced, and the reaction was allowed to proceed for 5 min. Subsequently, an ice bath was employed to lower the temperature to 45 °C. Following this, 20 mL of anhydrous ethanol was added, and the resulting mixture underwent centrifugation at 12,000 rpm for 10 min. The supernatant was collected, and n-octane was added to disperse the nanoparticles at a concentration of 80 mg/mL. Similarly, for the 1-Dodecanethiol (DT)-ligand-assisted synthesis method, 2.2532 g of Pb(NO_3_)_2_ and 1.6560 g of Na(DDTC) were mixed with 11.5 mL of DT. The mixture underwent degassing at 50 °C for one hour before reaching experimental temperatures (120 °C, 150 °C, 180 °C, 210 °C, and 240 °C). Following temperature adjustment to 45 °C with an ice bath, 20 mL of anhydrous ethanol was added, and the reaction continued for 3 min. Subsequently, the same centrifugation method was employed, and the supernatant was collected and mixed with n-Octane. A schematic diagram illustrating this synthesis process is depicted in Figure 1.

The PbS nanoparticles underwent X-ray diffraction (XRD) analysis using a Cu-Kα radiation source (λ = 0.1542 nm) with an X-ray diffractometer (D2 PHASER XE-T, BRUKER, Billerica, MA, USA). Morphological characterization was performed via transmission electron microscopy (TEM) (JEM-2100 plus, JEOL, Tokyo, Japan). Photoluminescence (PL) measurements were conducted using an LSLS-QY instrument with an excitation wavelength of 785 nm. The UV/VIS/NIR spectra were determined using a V-770 JASCO UV/VIS/NIR spectrophotometer (JASCO North America, Easton, MD, USA) equipped with a deuterium lamp (190~350 nm) and halogen lamp (330 nm~2700 nm) as excitation light sources. Contact angle measurements were conducted using a DMo-602 apparatus. Two solutions containing PbS nanoparticles, synthesized with ODE and DT as precursors, were dispersed in DMF. These solutions were then applied onto plain glass slides to facilitate contact angle measurements. The Fourier-transform infrared spectroscopy (FTIR) analysis was carried out using a JASCO FT/IR-4600 spectrometer (JASCO North America, Easton, MD, USA) without an attenuated total reflection (ATR) unit. Atomic Force Microscopy (AFM) was conducted using a Bruker/Innova instrument. Dynamic Light Scattering (DLS) measurements were performed with an ELSZ-2000 instrument (Otsuka, Tokyo, Japan).

## 3. Results and Discussion

**Figure 2:**
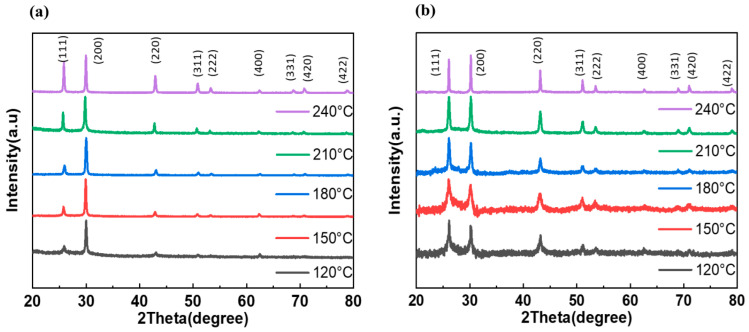
XRD patterns of the PbS nanoparticles prepared at various temperatures using (**a**) ODE-OAm and (**b**) DT.

The X-ray diffraction patterns of PbS nanoparticles obtained from ODE-OAm and DT ligand samples are illustrated in Figure 2a,b, respectively. Notably, diffraction peaks are observed at 2θ values of 25.80°, 29.92°, 42.87°, 50.78°, 53.29°, 62.40°, 68.76°, 70.79°, and 78.76°, corresponding to the (1 1 1), (2 0 0), (2 2 0), (3 1 1), (2 2 2), (4 0 0), (3 3 1), (4 2 0), and (4 2 2) planes of the face-centered cubic structure of PbS formation observed in both samples, thereby confirming JCPDS Card No. 65-2935 [26]. Interestingly, the PbS-DT treatment shows preferential orientation of the (111) planes within the temperature range of 120 °C–240 °C. In contrast, in the ODE-OAm ligand-assisted method, a notable crystal orientation peak at 25.80° was observed at 240 °C, suggesting that elevated temperatures are required to grow along the (111) orientation. This finding diverges from the behavior observed in DT-assisted PbS nanoparticles, where temperatures ranging from 120 °C to 240 °C demonstrated that PbS nanoparticles grew along the (111) direction even at lower temperatures, as depicted in Figure 2b.

The mechanism involves nucleation, where growth initiates from the rock-salt-like seed crystal of PbS particles. These PbS seeds are tetradecahedrons, and after the condensation reaction, they transform into cubes grown along the (111) direction. When utilizing ODE-OAm ligands, the seed crystal transforms into a cubic structure with (111) planes; however, the (200) plane is more preferential. Moreover, the variation in PbS morphology could potentially arise from the robust selective bonding of DT molecules onto the PbS nanoparticles, thereby exerting a significant influence on their shapes [27]. In this case, crystal growth perpendicular to the (111) plane is preferred, as it results in different morphologies from the OAm ligands. This underscores the tendency of thiol-treated molecules to favor the (111) planes and suggests their effectiveness in nanoparticle preparation [28]. In addition, substituting the long, insulating ODE-OAm capping molecules with shorter DT ligands produces hexagonal nanoparticles with excellent crystallinity and morphology. Similarly, the shorter ligand undergoes a condensation reaction, thereby enhancing conductivity and facilitating electron transfer. This promotes electron coupling between nanoparticles, particularly within the optimal temperature range of 200 °C [24,29]. Moreover, the hexagonal shape of PbS nanoparticles is often preferred over cubic shapes due to their enhanced properties, including higher surface-to-volume ratios and improved packing abilities, which can lead to enhanced reactivity on the nanoparticle surface.

**Figure 3:**
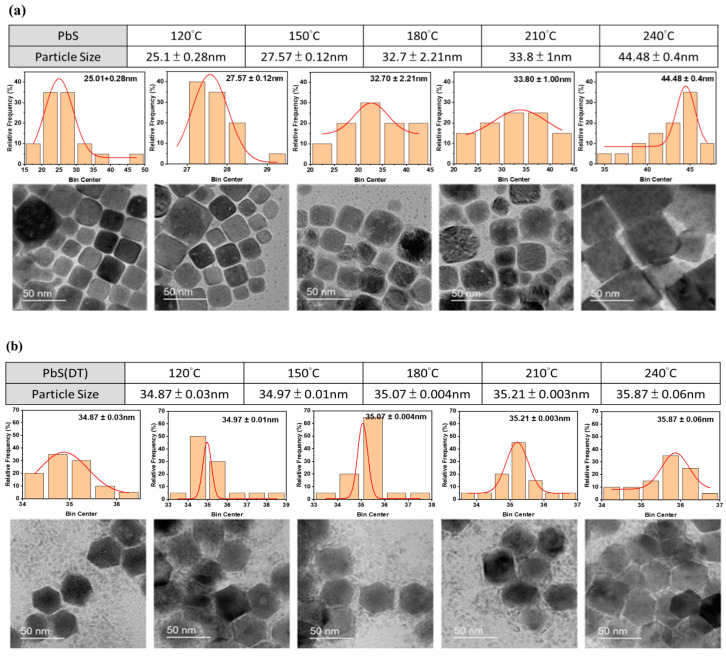
The various sizes of PbS nanoparticles along with their corresponding TEM images, demonstrating different synthesis processes: (**a**) with ODE-OAm and (**b**) DT.

The TEM images reveal the varied sizes of PbS nanoparticles, ranging from 25.1 nm to 44.48 nm, synthesized using ODE-OAm at temperatures of 120 °C, 150 °C, 180 °C, 210 °C, and 240 °C. Notably, at 240 °C, a larger particle size of 44.48 nm was observed, displaying a larger cubic shape compared to other temperatures (Figure 3a). Consequently, in the DT-capped PbS nanoparticles, a slight increase in particle size was observed with increasing temperature. However, all particles exhibited sizes between 34.87 nm and 35.87 nm and displayed a hexagonal shape (Figure 3b), contrasting with the cubic shape. In this case, minimal variation in the size of the nanoparticles was observed, contrasting with the significant variation observed with OAm ligands, as depicted in the upper portion of Figure 3a. It should be noted that ODE in OAm facilitates the production of monodisperse PbS nanoparticles and can serve as a co-reducing agent. The formation of cubic-shaped structures, consistent with slower metal deposition, was observed. Besides stabilizing agents, temperature also plays a crucial role in shaping outcomes; lower temperatures tend to yield fewer nanocubes with smaller nanoparticle sizes. Conversely, higher temperatures lead to the formation of larger nanoparticles with irregular cubic shapes [29]. However, when PbS nanoparticles are capped with DT, the stronger binding affinity and effective ligand–shell interactions provided by DT stabilize the nanoparticles. This suggests that the particles are less prone to dissolution and redeposition onto larger hexagonal particles, resulting in a consistent particle size distribution across various temperatures.

**Figure 4:**
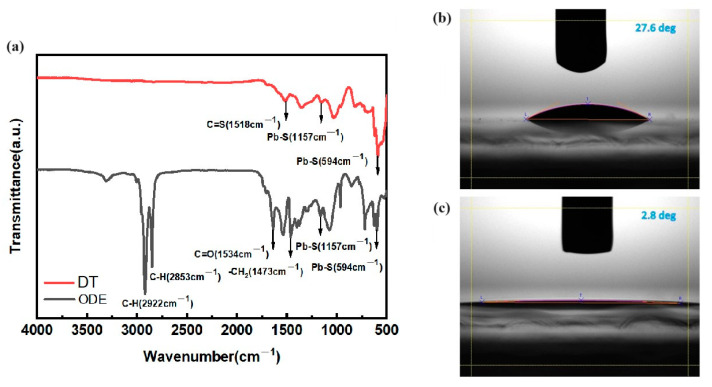
(**a**) FTIR spectra of ODE-OAm and DT ligands, along with the contact angles of PbS nanoparticles prepared using different synthesis processes: (**b**) with ODE-OAm and (**c**) DT.

The FT-IR spectra reveal distinctive features: A prominent stretching vibration at 1642 cm^−1^, corresponding to the carbonyl group of C=O, is indicative of the presence of OAm on the PbS nanoparticles, as illustrated in Figure 4a [30]. Additionally, the antisymmetric C–H stretching vibrations at 2853 cm^−1^ and 2922 cm^−1^ suggest the involvement of OAm molecules. The peaks observed in these range are attributed to the C-H bonds present in the butylamine and oleic acid ligands [31]. In the thiol-treated samples, these ligands are replaced by thiol-functionalized ligands, which are not observed in the FTIR spectrum (Figure 4a). In addition, the chemical bond between PbS and DT is affirmed by the presence of the C–S vibration at 1518 cm^−1^, signifying the co-capping of DT molecules on the PbS nanoparticles [21]. Notably, characteristic peaks at 1157 cm^−1^ and 594 cm^−1^, attributed to heteropolar diatomic molecules of lead sulfide, are observed in both spectra, as depicted in Figure 4a. Moreover, the absence of C=O stretching vibrations at 1534 cm^−1^ in the DT ligands confirms that the nanoparticles were not capped with oleic acid [32]. The FTIR spectra of the OAm and DT ligands used in the synthesis of PbS nanoparticles at various temperatures are presented in Supporting Information Figure S1. At 250 °C, the spectra reveal distinct chemical interaction states between the molecules in both ligand preparations.

The results of the contact angle measurement indicate that the choice of different ligands for capping the nanoparticles significantly influences the stability of the materials under moist conditions. In general, the wettability of the specific surface, whether hydrophilic or superhydrophilic, is determined based on the contact angle, with greater than 10° (less than 90°) indicating hydrophilicity and less than 10° indicating superhydrophilicity [33]. Water contact angle measurements of PbS surfaces with ODE-OAm and DT, prepared at a temperature of 240 °C, are depicted in Figure 4b,c. When PbS nanoparticles are capped with ODE-OAm ligands, there is a pronounced hydrophilicity with a contact angle of 27.6° (Figure 4b). After passivation with DT ligands, a shift towards superhydrophilicity is observed with a contact angle of 2.8° due to the presence of thiol groups. The surface roughness of ODE-OAm is relatively high, but it can be improved by replacing it with DT, as confirmed by AFM images presented in Supporting Information Figure S2. This may be attributed to the higher water repellency obtained by the larger surface roughness of the PbS nanoparticles with the addition of ODE-OAm compared to the DT-capped PbS nanoparticles.

**Figure 5:**
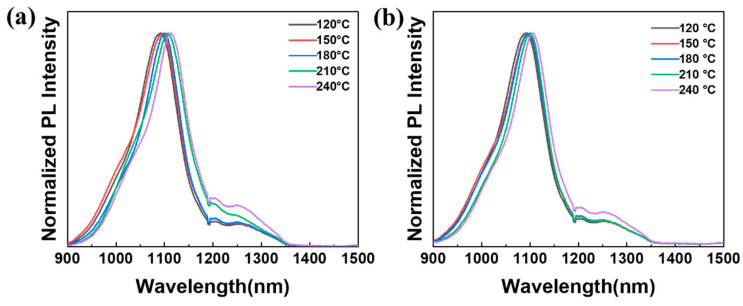
PL spectra of PbS nanoparticles prepared under varying temperatures using (**a**) ODE-OAm and (**b**) DT ligands.

Figure 5 displays the PL spectra of various ligands, including ODE-OAm and DT, in conjunction with PbS samples synthesized at temperatures ranging from 120 °C to 240 °C. Notably, the PL spectral intensity reveals a prominent peak centered around 1100 nm for nanoparticles prepared with both ODE-OAm and DT ligands. The UV-vis absorption spectra of both ligands are located between 950 nm and 1000 nm, with no significant changes observed, as shown in Supporting Information Figure S3. In addition, the PL measurements illustrate observable NIR emissions for all the nanoparticles when varying the temperature. Significantly, the nanoparticles prepared with DT at 240 °C demonstrated a quantum-confinement regime, indicating a high quantum-confinement effect in these nanoparticles [29,34]. The influence of the capping DT ligand on PbS nanoparticles reveals that the excitonic peaks of the nanoparticles at 240 °C are red-shifted compared to those at 120 °C. We propose that this red shift occurs during the condensation reaction between the thioacetate groups at elevated temperatures. This trend is also similar to the synthesis of PbS nanoparticles with ODE ligands at high temperatures.

## 4. Conclusions

In our synthesis method, the combined effect of capping ligands and solvents played an effective role in obtaining PbS nanoparticles of different sizes and shapes. The use of ODE-OAm as the capping ligand and solvent produced cubic-shaped nanoparticles, while DT alone yielded hexagonal-shaped nanoparticles. As demonstrated, the DT ligand acts as a passivator during the synthesis process of PbS nanoparticles, facilitating the transformation from cubic to hexagonal shape. Furthermore, replacing the long, insulating ODE-OAm capping molecules with shorter DT ligands promotes the (111) orientation, suggesting their effectiveness in nanoparticle preparation. Notably, at 240 °C, a larger particle size of 44.48 nm was observed with ODE-OAm ligands, showcasing a larger cubic shape compared to other temperatures. In contrast, with DT-capped PbS nanoparticles, a slight increase in particle size was observed with increasing temperature. Additionally, it contributes to the superhydrophilic nature, with an angle of 2.8° observed for the nanoparticles synthesized using DT, compared to those with ODE-OAm ligands with a contact angle of 27.6°. Moreover, we can enhance near-infrared photoluminescence (~1100 nm) by adjusting the temperature of the DT-capped PbS. Consequently, the influence of the DT-capped PbS nanoparticles reveals that the excitonic peaks of the nanoparticles at 240 °C are red-shifted due to the high quantum-confinement effect compared to the lower temperature. The results confirm that the hexagonal-shaped PbS nanoparticles can be candidates for IR photoreactors and bioimaging applications.

## Data Availability

Data are contained within the article and Supplementary Materials.

## Supplementary Materials

The following supporting information can be downloaded at: https://www.mdpi.com/article/10.3390/ma17102380/s1. Figure S1. FTIR ((a) ODE and (b) DT) spectra of PbS nanoparticles prepared with different temperatures. Figure S2. AFM images of PbS nanoparticles prepared with ODE-OAm and DT. Figure S3. Absorption spectra of PbS nanoparticles prepared with ODE-OAm and DT ligand with different temperatures.
