# Supplementary material for: Stable Near-Infrared Photoluminescence of Hexagonal-Shaped PbS Nanoparticles with 1-Dodecanethiol Ligands
Source: Materials (Basel). 2024 May 16;17(10):2380. doi: 10.3390/ma17102380 (PMC11123402; doi:10.3390/ma17102380)
Supplement: Supplementary file 1 [file materials-17-02380-s001.zip › materials-2959907-supplementary.pdf]

Supporting Information

Stable Near-Infrared Photoluminescence of Hexagonal-Shaped PbS Nanoparticles with 1-Dodecanethiol Ligands

Tsair-Chun Liang [1], Hsin-Yu Su [1], Kasimayan Uma [2], Sih-An Chen [2,3,4], Zhi-Chi Deng [3], Tzung-Ta Kao [1], Chun-Cheng Lin [4,*] and Lung-Chien Chen [3,*]

[1] Institute of Photonics Engineering, National Kaohsiung University of Science and Technology, Kaohsiung 824, Taiwan
[2] Organic Electronics Research Center, Ming Chi University of Technology, New Taipei City 243, Taiwan
[3] Department of Electro-Optical Engineering, National Taipei University of Technology, Taipei 106, Taiwan
[4] Department of Mathematic and Physical Sciences, General Education Center, R.O.C. Air Force Academy, Kaohsiung 820, Taiwan
* Correspondence: cclincafa@gmail.com (C.-C.L.); ocean@ntut.edu.tw (L.-C.C.)

S1

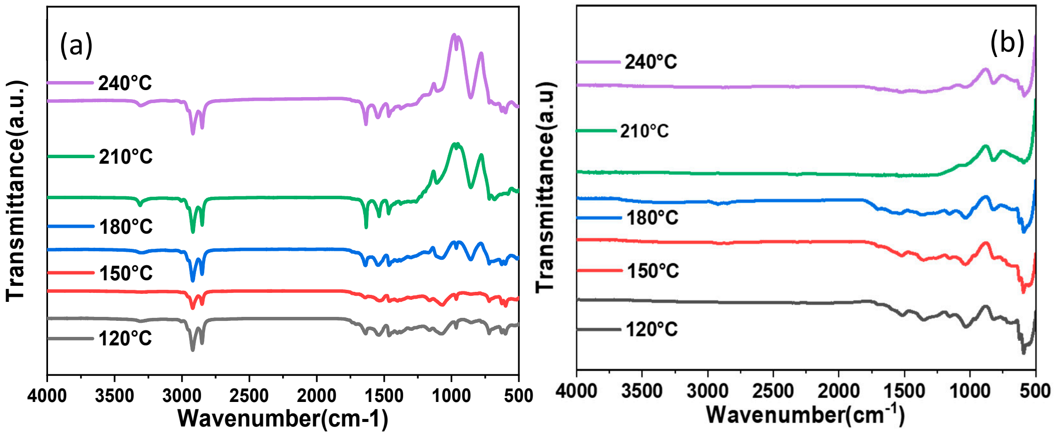

Figure S1. FTIR ((a) ODE and (b) DT) spectra of PbS nanoparticles prepared with different temperatures.

S2

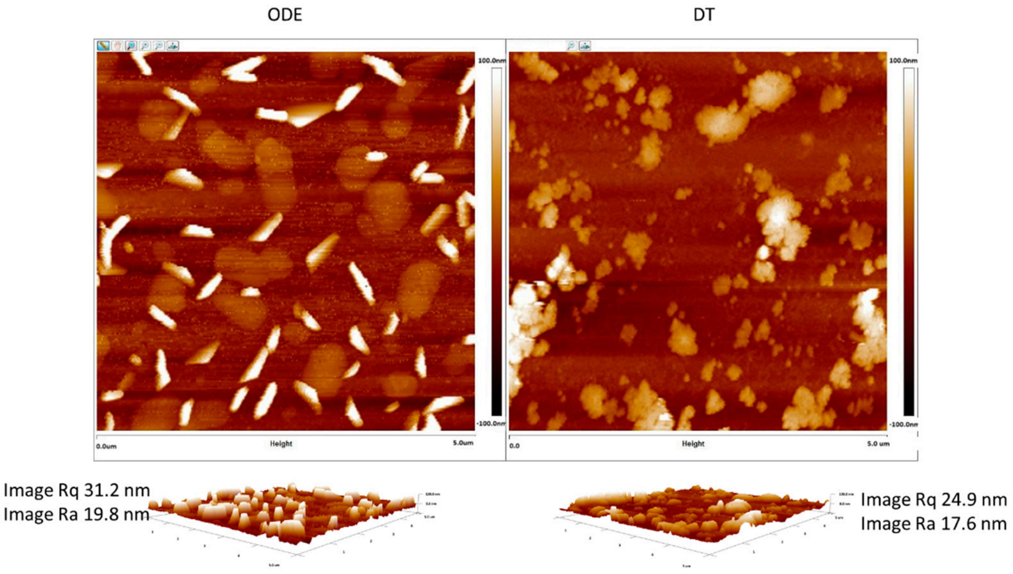

Figure S2. AFM images of PbS nanoparticles prepared with ODE-OAm and DT.

S3

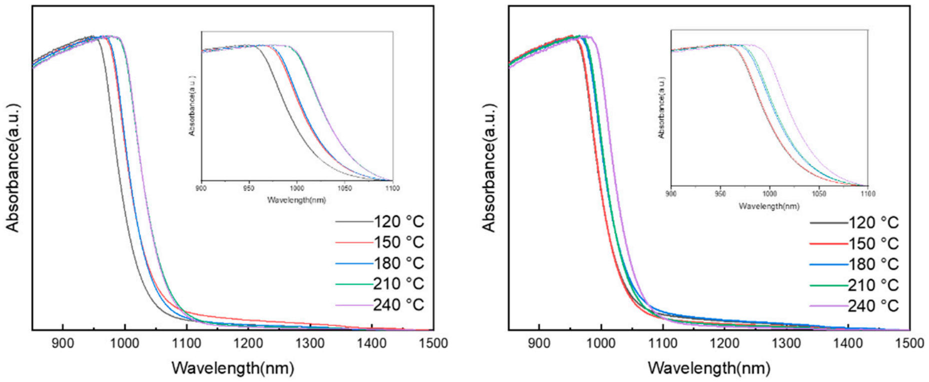

Figure S3. Absorption spectra of PbS nanoparticles prepared with ODE-OAm and DT ligand with different temperatures.
